# Comprehensive RNA-Seq Analysis of Human Osteoclast Function in Response to *Bothrops moojeni* Venom Fractions: Pathways of Bone Resorption and Cytoskeletal Disruption

**DOI:** 10.3390/toxins17070358

**Published:** 2025-07-19

**Authors:** Fernanda D’Amélio, Hugo Vigerelli, Rodrigo Pinheiro Araldi, Isabel de Fátima Correia Batista, Daniel Carvalho Pimenta, Irina Kerkis

**Affiliations:** 1 Postgraduate Program in Structural and Functional Biology, UNIFESP, São Paulo 04023-062, Brazil; 2 Centre of Excellence in New Target Discovery (CENTD), Butantan Institute, São Paulo 05503-900, Brazil; hugo.barros@butantan.gov.br (H.V.); isabel.batista@butantan.gov.br (I.d.F.C.B.); 3 Laboratory of Genetics, Butantan Institute, São Paulo 05503-900, Brazil; rodrigo.pinheiro.araldi@gmail.com; 4 Laboratory of Ecology and Evolution, Butantan Institute, São Paulo, 05503-900, Brazil; dcpimenta@butantan.gov.br

**Keywords:** *Bothrops moojeni* venom, osteoclast function, RNA-seq analysis, bone resorption, cytoskeletal disruption, snake venom fractions

## Abstract

This study investigated the effects of *Bothrops moojeni* (*B. moojeni*) venom and its high- (HMM) and low-molecular mass (LMM) fractions on human osteoclast (OC) differentiation and function in vitro, aiming to identify novel therapeutics for bone disorders. Venom preparations were applied at 5 µg/mL (crude venom and HMM) or 1 µg/mL (LMM) from day 4 of peripheral blood mononuclear cell (PBMC) differentiation through terminal OC formation, enabling evaluation across early differentiation, fusion, and maturation stages. RNA sequencing revealed 7793 genes common to all experimental groups, with unique gene expression signatures of 149 (control), 221 (HMM), 248 (crude venom), and 60 (LMM) genes, reflecting distinct molecular responses. The negative control PBMC group exhibited 1013 unique genes enriched in immune-related pathways, consistent with their undifferentiated state. Crude venom induced the broadest transcriptional modulation, upregulating key fusion (CD47) and resorption (CTSK) genes, and altering markers of OC differentiation. The HMM fraction predominantly influenced inflammatory and osteoclastogenic pathways, notably TNF and NF-κB signaling, while the LMM fraction selectively regulated fusion-related genes (e.g., CD44) and immune pathways, indicating targeted modulation of OC activity. Cytokine profiling showed that crude venom and HMM suppressed osteoclastogenic cytokines such as IL-1β and IL-6, supporting their potential use in inflammatory bone diseases. Pathway enrichment analyses confirmed these differential effects on immune response and bone resorption mechanisms. Together, these results demonstrate that *B. moojeni* venom and its fractions differentially impact OC biology, with crude venom exerting broad effects and HMM and LMM fractions offering more specific modulation. Future studies will isolate bioactive components and assess therapeutic efficacy in animal models of osteoporosis and rheumatoid arthritis.

## 1. Introduction

Osteoclasts (OCs) are specialized multinucleated cells responsible for bone resorption, playing a crucial role in maintaining bone homeostasis. Their differentiation is a complex and tightly regulated process orchestrated by key factors, including macrophage colony-stimulating factor (M-CSF) and receptor activator of nuclear factor kappa-B ligand (RANKL). M-CSF promotes the survival and proliferation of precursor cells, while RANKL triggers their differentiation into mature OC [1]. Precursor cells express the RANK receptor, and upon RANKL binding, activate signaling pathways such as NF-kB, MAPK, and c-Fos, which are essential for OC maturation and fusion. The fusion of precursor cells into multinucleated OCs is mediated by proteins like DC-STAMP and OC-STAMP. These mature OCs then form an actin ring, which enables bone resorption through the release of acids and proteolytic enzymes. Dysregulation of this process can lead to various bone-related diseases, such as osteoporosis, characterized by excessive bone resorption [2].

The venom of *Bothrops moojeni*, a venomous snake native to Brazil, has been shown to exhibit potent bioactivity, with diverse effects on cellular processes, including inflammation, immune modulation, and cellular signaling [3,4,5,6]. *B. moojeni* venom has a complex and diverse composition that remains largely underexplored. While traditionally studied for its toxic effects, recent research highlights its components as a rich source of novel bioactive molecules with promising pharmacological and biotechnological applications [4]. Comprehensive analyses of the venom have identified (i) 20 classes of venom components, with metalloproteases being the most abundantly expressed, followed by serine proteases and phospholipases, and (ii) 33 full-length toxin amino acid sequences not previously reported in *B. moojeni* venom. These include one cysteine-rich secretory protein (Moojin), two hyaluronidases (BmooHyal-1 and BmooHyal-2), and one three-finger toxin (Bmoo-3FTx) [7]. Moreover, recent studies have suggested that the venom may influence OC biology, particularly through its impact on cytokine production, resorptive activity, and cytoskeletal organization. However, the specific molecular mechanisms underlying these effects remain poorly understood [8].

RNA sequencing (RNA-Seq) has emerged as a powerful tool for investigating the transcriptomic landscape of cells exposed to complex stimuli, allowing for a comprehensive analysis of gene expression changes at a global level [9]. In this study, we utilized RNA-Seq to investigate the transcriptional responses of human OCs exposed to different fractions of *B. moojeni* venom: crude venom, high molecular mass (HMM), and low molecular mass (LMM). Our goal was to elucidate the molecular pathways involved in bone resorption and cytoskeletal disruption induced by venom exposure, focusing on gene expression profiles related to OC differentiation, fusion, and resorptive function [2,8].

This study aims to provide a deeper understanding of how venom-derived fractions influence human OC activity at the molecular level, offering insights into their therapeutic potential for bone metabolic disorders. By identifying key genes and signaling pathways modulated by these components, we seek to uncover novel molecular targets for the treatment of bone resorption-related diseases and to broaden our understanding of the regulatory impact of venom-derived bioactive molecules on OC biology.

Furthermore, in this study, the crude venom, HMM, and LMM fractions were added at concentrations of 5 µg/mL, 5 µg/mL, and 1 µg/mL, respectively, starting on day 4 of OC differentiation and maintained continuously throughout the entire differentiation period. This experimental design allowed us to assess the effects of these treatments across all stages of OC development—including early differentiation, cell fusion, and terminal maturation. By sustaining exposure to the venom and its fractions, we were able to comprehensively evaluate how these bioactive compounds influence osteoclastogenesis and functional activity over time.

## 2. Results

### 2.1. Venn Diagram Analysis Reveals Core and Treatment-Specific Transcriptional Signatures

The Venn diagram analysis provided a comprehensive overview of gene expression overlap and specificity among experimental conditions, enabling us to delineate core transcriptional programs from treatment-specific responses. Genes shared across all groups represent a common molecular signature, likely associated with basal OC differentiation or conserved cellular functions, while uniquely expressed genes point to the specific effects of each venom fraction.

**Figure 1:**
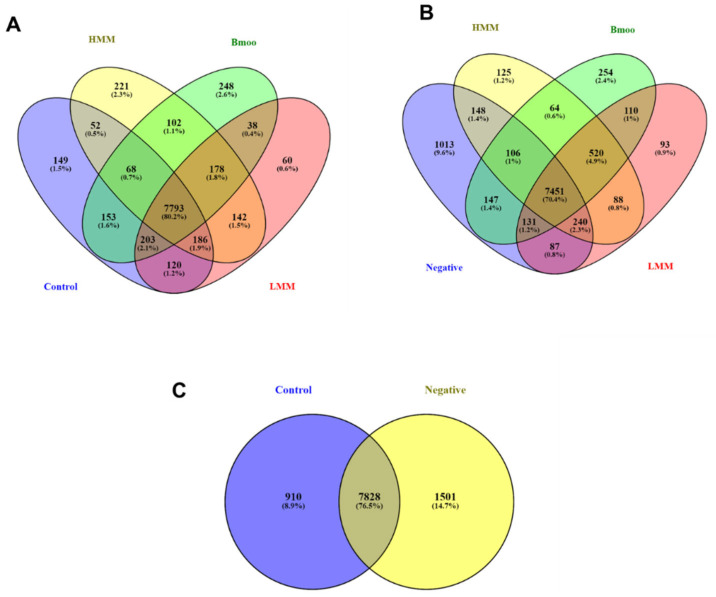
Venn diagram analysis of gene expression profiles across experimental groups. (**A**) Overlap of expressed genes across all groups, highlighting 7793 genes common to all conditions. Unique genes identified in the control group (149), HMM fraction-treated group (221), crude venom-treated group (248), and LMM fraction-treated group (248). (**B**) Analysis of genes shared across all groups, excluding the negative control, showing 7451 common genes. Unique genes are indicated for the negative control (1013), HMM-treated (125), crude venom-treated (254), and LMM-treated (93) groups. (**C**) Comparative gene expression between negative control (PBMCs) and positive control (PBMCs differentiated into OCs without venom treatment). The 1013 unique genes identified in PBMC cultures reflect their undifferentiated immune cell state, with enrichment in classical immune pathways, consistent with the expected immune-like phenotype in the absence of osteoclastogenic stimulation.

At all 7793 genes were found to be common across all experimental groups (Figure 1A). Additionally, 149 genes were uniquely expressed in the control group, 221 in the high molecular mass (HMM) fraction, 248 in the crude venom, and 60 in the low molecular mass (LMM) fraction. Several overlapping subsets among treatment combinations were also identified, underscoring shared regulatory pathways and potential synergistic effects between venom components.

A second analysis focusing on the negative control (Figure 1B)—representing unstimulated PBMCs—revealed 7451 genes shared across all groups. The negative control showed 1013 unique genes, with other exclusive signatures observed in the HMM (125), crude venom (254), and LMM (93) groups. Overlaps among combinations of treatment and control groups highlight the complex transcriptional remodeling induced by each condition relative to the basal state.

The comparison between negative control (PBMCs alone) and positive control (PBMCs induced to differentiate into OCs without venom exposure) helped isolate gene expression changes directly linked to osteoclastogenesis (Figure 1C). This baseline is essential for interpreting the transcriptional impact of venom fractions, ensuring that observed differences are not merely due to differentiation itself but are specifically attributable to the treatments.

This multi-level Venn diagram approach not only maps the transcriptional landscape of venom fraction exposure but also supports mechanistic insight into OC modulation and provides a rational framework for identifying candidate genes for further validation as therapeutic targets or biomarkers.

### 2.2. Impact of Venom Fractions on Osteoclastogenesis: Gene Expression Analysis of Cytokines, Differentiation, Fusion, and Resorption Pathways

The heatmap technique was employed to analyse the clusters of interest, including genes directly involved in processes such as resorption, differentiation, fusion, osteoclastogenic and anti-osteoclastogenic cytokines, inhibitory pathways of osteoclastogenesis, and canonical and non-canonical differentiation pathways. This analysis enabled the identification of distinct gene expression patterns linked to these processes, offering a comprehensive view of the transcriptional changes associated with different treatments and their interactions in osteoclastogenesis.

**Figure 2:**
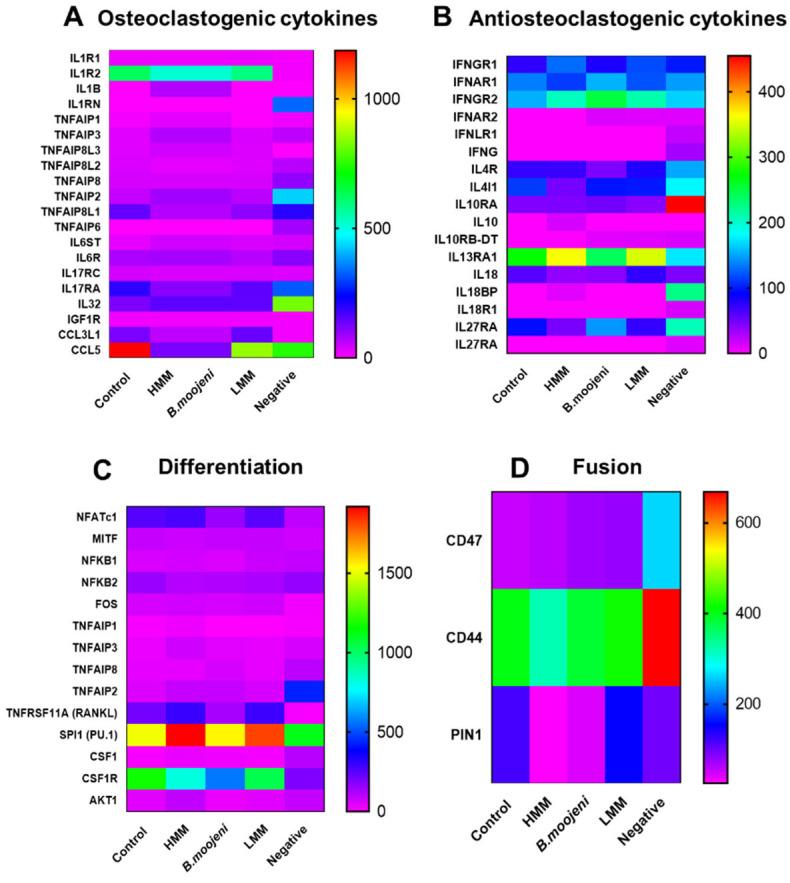
Heatmap cluster analysis of gene expression across control and experimental groups. (**A**) Expression patterns of osteoclastogenic cytokines in control and venom-treated groups. (**B**) Expression profiles of anti-osteoclastogenic cytokines across all groups. (**C**) Differential expression of genes associated with OC differentiation in control versus treated samples. (**D**) Gradual upregulation of the fusion-related gene *CD47* observed in crude venom, HMM, and LMM fraction treatments relative to the positive control.

The analysis of osteoclastogenic and anti-osteoclastogenic cytokines (Figure 2A,B) revealed distinct effects of crude venom, high molecular mass (HMM), and low molecular mass (LMM) treatments. For osteoclastogenic cytokines (Figure 2A), crude venom treatment reduced IL1R2, TNFAIP8L1, and IL17Ra expressions, suggesting suppression of osteoclastogenesis and inflammatory pathways. Additionally, crude venom lowered CCL3 and CCL5 expression, indicating a reduction in immune cell recruitment. Similarly, HMM treatment decreased IL1R2, TNFAIP8L1, and IL17Ra, inhibiting OC differentiation. CCL5 expression was significantly reduced, further suggesting suppression of immune responses and osteoclastogenesis. In contrast, LMM treatment showed more balanced IL1R2 expression, comparable to the positive control, with moderate reductions in TNFAIP8L1, IL17Ra, and CCL5 expression.

Regarding anti-osteoclastogenic cytokines (Figure 2B), both crude venom and HMM treatments led to an increase in IL-32 expression, suggesting upregulation of inflammatory pathways that may enhance OC differentiation. LMM treatment also resulted in higher IL-32 expression than the positive control, though the effect was less pronounced than in the crude venom and HMM treatments. Therefore, while crude venom and HMM treatments suppress osteoclastogenic pathways, they enhance anti-osteoclastogenic cytokines like IL-32, whereas LMM treatment exhibits a more balanced modulation of cytokines.

In the analysis of differentiation-related genes (Figure 2C), NFATc1 expression was decreased following crude venom treatment compared to the positive control. RANKL expression increased in both HMM and LMM treatments but decreased in the crude venom group. PU.1 expression was upregulated in both HMM and LMM treatments. On the other hand, CSF1R was downregulated in the HMM and crude venom groups relative to the control.

For fusion-related genes (Figure 2D), CD47 expression gradually increased across all crude venom, HMM, and LMM treatments relative to the positive control. CD44 showed a slight decrease in the HMM-treated group compared to the control. PIN1 expression was reduced in both HMM and crude venom treatments.

**Figure 3:**
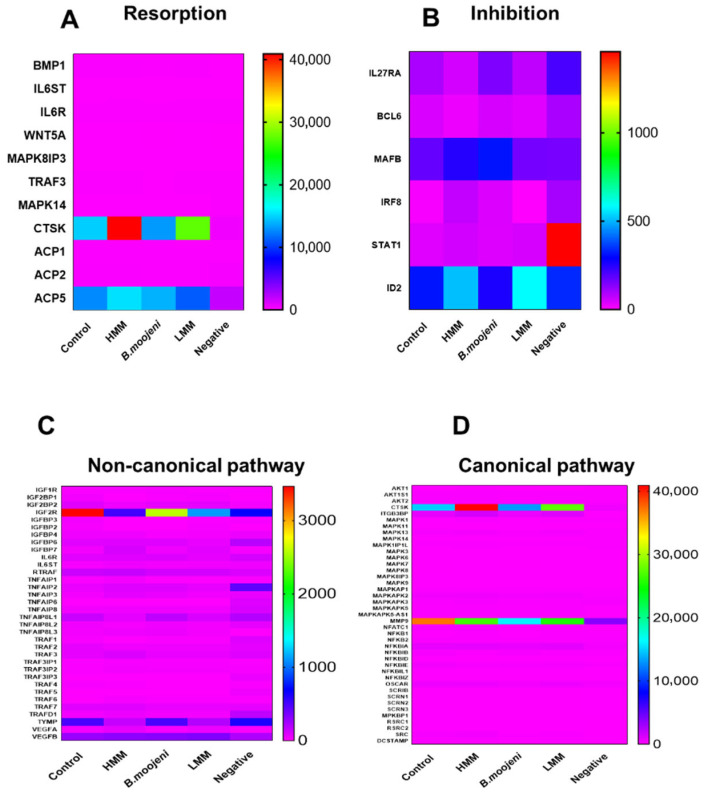
Heatmap cluster analysis of gene expression related to osteoclast function across control and experimental groups. (**A**) Expression profiles of genes involved in resorption processes in control and treated groups. (**B**) Differential expression of genes associated with inhibition pathways. (**C**) Analysis of non-canonical pathway gene expression across all groups. (**D)** Expression patterns of canonical pathway genes in control versus experimental groups.

In the analysis of resorption-related genes (Figure 3A), CTSK expression was elevated in all treatments (HMM, LMM, and crude venom) compared to the control. ACP5 expression increased in the HMM and crude venom groups but decreased in the LMM-treated group relative to the positive control.

Regarding inhibition-related genes (Figure 3B), IL27RA showed a slight decrease in both HMM and LMM treatments, with a slight increase in the crude venom group. MAFB was upregulated in the HMM and crude venom groups but downregulated in the LMM group. IRF8 expression was increased in the HMM and crude venom-treated groups, while it decreased in the LMM group. STAT1 expression was slightly elevated in HMM and LMM treatments compared to the positive control, and ID2 expression increased in both HMM and LMM treatments, while the crude venom group remained similar to the control.

In the non-canonical pathway (Figure 3C), the IGF2R gene exhibited a marked decrease in expression in the HMM, LMM, and crude venom-treated groups compared to the positive control. Similarly, the TYMP gene was downregulated in both HMM and LMM treatments relative to the positive control. In the canonical pathway (Figure 3D), significant modulation was observed in the CTSK gene, with noticeable upregulation in both HMM and LMM treatments. Additionally, MMP9 expression was reduced in all treatment groups (crude venom, HMM, and LMM) relative to the control.

### 2.3. Molecular Pathways Regulating Osteoclast Differentiation, Fusion, and Function: Insights from Venom Fractions and STRING Analysis

**Figure 4:**
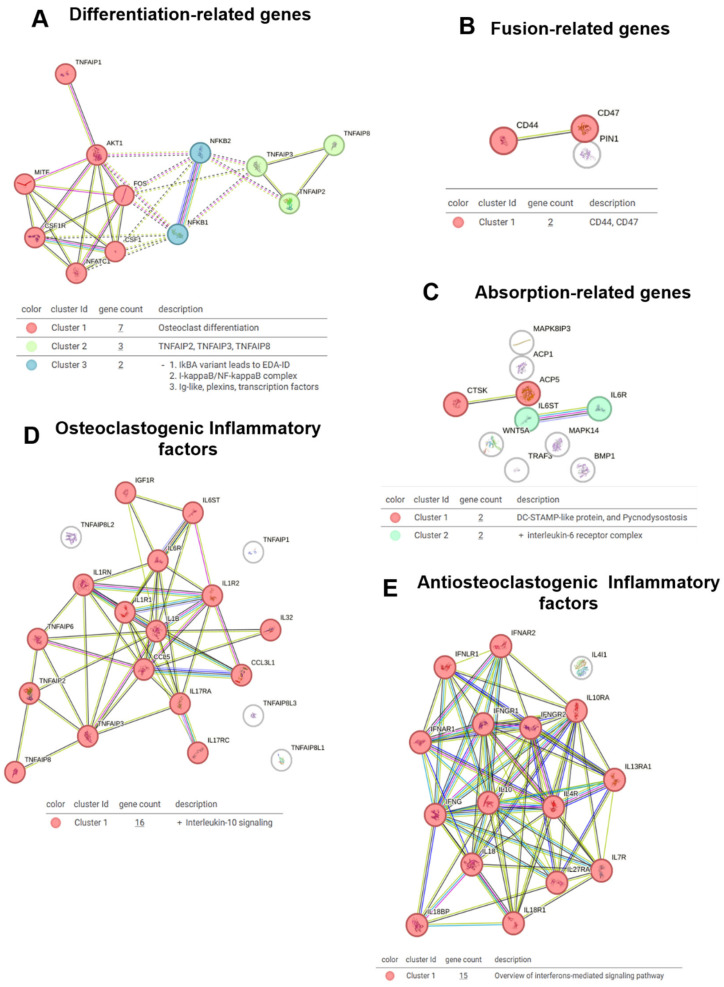
STRING network analysis of key gene clusters involved in osteoclast biology. (**A**) Network of genes related to osteoclast differentiation. (**B**) Interactions among fusion-related genes. (**C**) Network analysis of genes associated with bone resorption. (**D**) Osteoclastogenic inflammatory factors and their predicted interactions. (**E**) Anti-osteoclastogenic inflammatory factors and their functional associations.

The STRING analysis (SA) provided a comprehensive overview of molecular interactions related to OC differentiation, fusion, and function. The differentiation analysis (Figure 4A) revealed three distinct clusters: Cluster 1, which is related to classical OC differentiation pathways; Cluster 2, encompassing members of the TNF family, which are crucial for modulating inflammation and OC differentiation; and Cluster 3, which is associated with variants of the NF-kappa-B pathway and transcription factors, highlighting the complex regulatory mechanisms at play during OC differentiation.

The venom fractions, including crude venom, HMM (high molecular weight), and LMM (low molecular weight), influenced these differentiation-related clusters. Specifically, the HMM fraction induced broad inflammatory responses and OC differentiation, which likely contributed to the activation of Cluster 2 (TNF family) and Cluster 3 (NF-kappa-B variants). In contrast, the LMM fraction fine-tuned these effects, targeting specific immune pathways and modulating the differentiation process without inducing widespread inflammatory changes. This distinction underscores the nuanced role of venom components in OC differentiation.

In the fusion analysis (Figure 4B), a single cluster was identified that directly linked CD44 and CD47, molecules involved in OC precursor fusion. The LMM fraction played a crucial role in modulating this cluster, suggesting a targeted effect on the fusion process. This interaction underscores the integrated role of CD44 and CD47 in immune modulation and bone homeostasis. For resorption (Figure 4C), Cluster 1 was associated with DC-STAMP, a key molecule in OC fusion, while Cluster 2 was connected to the IL-6 receptor, a critical player in OC activation and bone resorption. The venom fractions further modulated these clusters, particularly DC-STAMP in the HMM fraction, which may enhance OC maturation and function.

Further investigation into osteoclastogenesis and inflammatory factors (Figure 4D) revealed Cluster 1 to be linked with IL-10 signaling, a well-known anti-osteoclastogenic factor. The HMM fraction likely contributed to the regulation of IL-10 signaling, as it induced broader inflammatory responses. For anti-osteoclastogenic inflammatory factors (Figure 4E), Cluster 1 was associated with interferon-mediated signaling, which also has a significant role in modulating osteoclastogenesis. The LMM fraction appeared to enhance this signaling pathway, fine-tuning osteoclastogenesis regulation and preventing excessive bone resorption.

**Figure 5:**
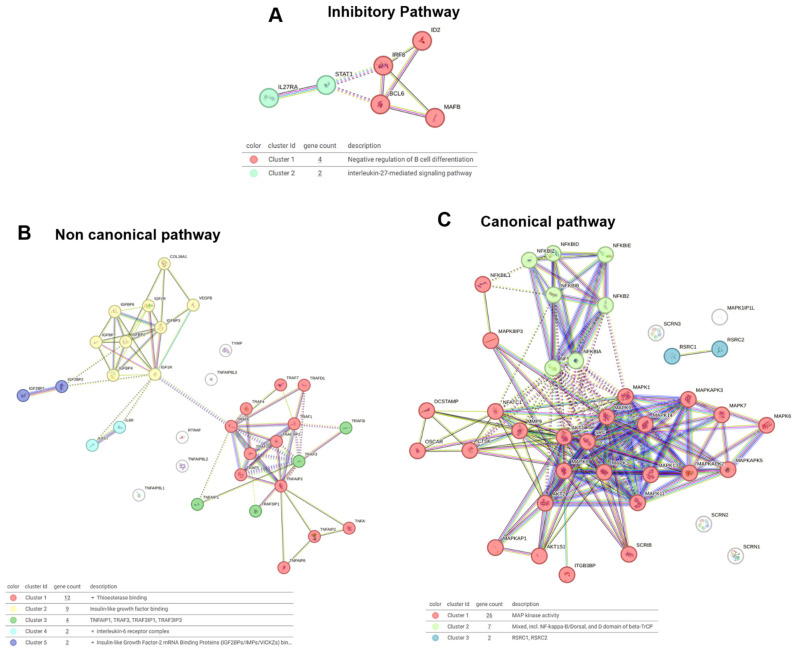
STRING cluster analysis of signaling pathways regulating osteoclast function. (**A**) Network of genes involved in inhibitory pathways. (**B**) Gene cluster associated with the non-canonical signaling pathway. (**C**) Interaction network of canonical pathway genes.

The inhibitory pathway analysis (Figure 5A) identified two clusters: Cluster 1, which is associated with the negative regulation of B cell differentiation, and Cluster 2, which is linked to IL-27-mediated signaling. The venom fractions, particularly LMM, may modulate these clusters, influencing immune responses and B cell activity, potentially affecting OC function and immune modulation in bone homeostasis.

In the non-canonical pathway analysis (Figure 5B), five clusters were identified: Cluster 1 was linked to thioesterase activity; Cluster 2 was associated with insulin-like growth factor binding; Cluster 3 included the TNF family; Cluster 4 was related to the IL-6 receptor complex; and Cluster 5 was associated with insulin-like growth factor mRNA binding. The HMM and LMM fractions both contributed to modulating these pathways, with HMM influencing broader immune signaling and LMM regulating specific growth factor interactions.

The canonical pathway analysis (Figure 5C) revealed three critical clusters: Cluster 1, which was linked to MAP kinase activity, Cluster 2, which involved NF-kappa-B signaling, and Cluster 3, associated with RSRC1 and RSRC2. The venom fractions, particularly the HMM fraction, likely activated NF-kappa-B signaling and MAP kinase activity, supporting the inflammatory response and OC differentiation. The LMM fraction appeared to modulate post-transcriptional regulation, influencing RSRC1 and RSRC2 to potentially regulate OC function at a molecular level.

The comparative analysis of *B. moojeni* venom fractions—crude venom HMM, and LMM—reveals both overlapping and distinct effects on OC biology. The HMM fraction induces broad inflammatory responses and activates classical osteoclastogenic pathways, particularly those involving TNF family members, NF-κB variants, and DC-STAMP. These effects enhance differentiation, fusion, and resorptive activity, making HMM especially relevant to inflammatory bone diseases such as osteoporosis and rheumatoid arthritis. In contrast, the LMM fraction fine-tunes immune modulation and selectively influences OC function by targeting fusion-related processes and signaling pathways involving cytokine and growth factor receptors. Although less inflammatory, dysregulation of LMM-mediated signaling may still promote bone loss by enhancing OC activity and disrupting the balance between bone resorption and formation. The key differences between HMM and LMM fractions are summarized in Table 1.

## 3. Discussion

Through transcriptomic profiling, we identified how exposure to distinct *B. moojeni* venom fractions—crude, HMM, and LMM—differentially influences OC function. Each fraction triggered unique transcriptional signatures that reflect modulation of pathways related to OC formation, maturation, and bone resorption. These findings underscore the complexity of venom–cell interactions and point to the therapeutic potential of specific venom components in regulating bone turnover. Complementary proteomic analyses previously conducted by our group [10] further support these observations, reinforcing the functional relevance of the transcriptional shifts and enhancing our understanding of the underlying molecular mechanisms.

Furthermore, in this model, human peripheral blood mononuclear cells provide a reservoir of OC precursors. Supplementation with M-CSF and RANKL not only promotes osteoclastogenesis but also maintains other monocyte-derived populations—such as macrophages—creating a mixed-cell culture that closely mirrors the in vivo OC niche. As a result, RNA-Seq profiling captures both the transcriptional signature of mature OCs and the contributions of unfused mononuclear and macrophage-like cells. This added complexity enhances the physiological relevance of the system, offering a more comprehensive understanding of OC biology within its native cellular microenvironment.

### 3.1. Impact of Venom Fractions on Osteoclastogenesis and Bone Resorption

Crude venom from *B. moojeni* contains a mixture of HMM and LMM components, each contributing to the modulation of osteoclastogenesis and inflammation. The HMM fraction exerts a broad-spectrum impact, primarily downregulating osteoclastogenic markers such as IL1R2 and TNFAIP8L1, and suppressing CCL5 expression, likely through mechanisms involving cytoskeletal disruption and immune modulation [11,12,13]. Additionally, the HMM fraction promotes inflammation by increasing IL-32 expression, which supports OC differentiation. The presence of phospholipases A2 (PLA2) and metalloproteinases (MMPs) within this fraction likely disrupts membrane integrity, further inhibiting critical signaling pathways associated with osteoclastogenesis [14].

In contrast, the LMM fraction exhibits a more selective and moderate influence on osteoclastogenesis. While it reduces the expression of osteoclastogenic markers such as TNFAIP8L1 and CCL5, its effect is less pronounced than that of the HMM fraction [15,16]. Notably, the LMM fraction enhances IL-32 expression, suggesting a mild pro-inflammatory effect that still facilitates OC differentiation, albeit without the extensive disruption seen with the HMM fraction. These findings imply that the smaller peptides or proteins in the LMM fraction selectively modulate cytokine signaling and immune responses, thereby promoting osteoclastogenesis without inducing the broader disruptions characteristic of the HMM fraction.

While bisphosphonates are widely used to inhibit bone resorption, they are associated with limitations such as poor tissue penetration, long-term skeletal retention, and adverse effects, including osteonecrosis of the jaw and atypical fractures [17]. In contrast, the LMW fraction—rich in disintegrins, knottins, and small peptides—modulated cytokine networks and osteoclastogenesis without widespread inflammation, suggesting superior tissue targeting and a lower risk of side effects. These findings support the potential of venom-derived compounds as refined therapies for osteoporosis, rheumatoid arthritis, and related diseases.

### 3.2. Core and Treatment-Specific Transcriptional Signatures

The Venn diagram analysis provided valuable insights into the transcriptional profiles induced by crude venom, HMM, and LMM treatments, revealing both shared and unique molecular responses. A common set of genes expressed across all treatment conditions reflects the fundamental processes of OC differentiation and core cellular functions, likely representing pathways essential for OC biology that are unaffected by the specific venom fractions. In contrast, treatment-specific gene expression patterns highlight distinct molecular mechanisms by which the venom fractions modulate OC behavior. The unique signatures observed in the HMM and LMM groups suggest that these fractions influence OC activity through separate signaling pathways, which could have important implications for bone health.

Furthermore, overlapping gene subsets between treatments indicate potential synergistic interactions between venom components. These results underscore the complexity of venom-induced changes in OC function and emphasize the need for further investigation into the molecular interactions between the venom fractions.

### 3.3. Venom Fraction-Induced Modulation of Osteoclastogenic and Anti-Osteoclastogenic Cytokines

Cytokine analysis revealed that both crude venom and the HMM fraction significantly suppressed osteoclastogenesis by downregulating key osteoclastogenic cytokines such as IL1R2, TNFAIP8L1, IL17Ra, and CCL5. These findings suggest that these venom fractions inhibit OC differentiation by interfering with the signaling pathways crucial for osteoclastogenesis [11,17]. Additionally, the reduced expression of immune cell recruitment markers, including CCL3 and CCL5, points to an anti-inflammatory effect, potentially mitigating bone resorption in inflammatory bone diseases. In contrast, LMM treatment induced a more balanced modulation of osteoclastogenesis, showing moderate reductions in TNFAIP8L1, IL17Ra, and CCL5, while maintaining IL1R2 expression at levels comparable to the positive control. This indicates that LMM fraction selectively targets specific immune pathways, modulating OC differentiation without promoting excessive inflammation [16].

Both crude venom and HMM treatments also resulted in increased IL-32 expression, an anti-osteoclastogenic cytokine that may suppress OC differentiation. The observed IL-32 upregulation alongside IL-1β/IL-6 suppression may reflect compensatory responses or selective pathway activation, highlighting the complex, context-dependent regulation of the cytokine network [18]. This underscores the dual nature of these venom fractions, modulating both pro- and anti-inflammatory pathways, which could provide therapeutic potential for controlling excessive bone resorption in diseases such as rheumatoid arthritis. In contrast, the more modest upregulation of IL-32 in LMM-treated OCs further supports its role in maintaining a more balanced immune modulation and bone homeostasis [15].

It is important to recognize that many cytokines involved in osteoclastogenesis exhibit pleiotropic and context-dependent roles, acting as both promoters and inhibitors depending on the microenvironment and cellular context [19]. For instance, IL-32, while generally considered anti-osteoclastogenic here, has also been reported to exert pro-inflammatory effects under certain conditions, contributing to disease progression in rheumatoid arthritis and other inflammatory states [20]. Similarly, chemokines like CCL5 and CCL3 can recruit immune cells that either promote bone resorption or support tissue repair, highlighting the complexity of cytokine networks in bone homeostasis [21]. This duality underscores the need for further studies to delineate the precise mechanisms and temporal dynamics by which venom fractions modulate these cytokine pathways to achieve therapeutic benefit without exacerbating inflammation.

### 3.4. Effects on Differentiation, Fusion, and Resorption Pathways

The analysis of differentiation-related genes revealed that crude venom suppressed NFATc1 expression, a key transcription factor for osteoclastogenesis, indicating that this venom fraction impairs OC differentiation [18,19]. In contrast, both HMM and LMM treatments upregulated RANKL expression, a critical factor for OC differentiation, suggesting that these fractions promote osteoclastogenesis. Additionally, the differential regulation of PU.1 expression, which is essential for OC precursor differentiation, further supports this conclusion [20,21].

Regarding fusion-related genes, CD47 expression was elevated across all treatment groups, with HMM and LMM showing more pronounced increases. CD47 plays a vital role in OC precursor fusion, and its upregulation suggests that these venom fractions facilitate OC precursor fusion, a key step in osteoclastogenesis. Notably, the slight decrease in CD44 expression in the HMM group may indicate more nuanced regulation of fusion markers by specific venom fractions [22].

In terms of resorption, all venom fractions upregulated CTSK expression, a hallmark of OC resorptive activity. This suggests that, despite their effects on differentiation, these venom fractions may also enhance OC function [23,24]. However, the differential regulation of ACP5 expression [25,26] between the venom fractions—HMM and crude venom, promoting increased expression, while LMM suppresses it—indicates that LMM may regulate OC resorption more finely, potentially preventing excessive bone resorption in certain conditions.

The differential regulation of fusion-related genes likely reflects the distinct composition and bioactivity of each venom fraction, potentially involving effects on membrane dynamics or fusion signaling pathways.

### 3.5. Canonical and Non-Canonical Pathways

The analysis of canonical and non-canonical pathways provided further insights into how venom fractions influence OC biology at the molecular level. The HMM fraction strongly modulated NF-kappa-B signaling and MAP kinase activity, both critical for OC differentiation and inflammatory responses, suggesting that HMM induces broad inflammatory responses that promote OC differentiation and bone resorption. In contrast, the LMM fraction influenced NF-kappa-B signaling to a lesser extent, suggesting a more refined regulatory role in OC function [27,28].

Non-canonical pathways, including those related to IGF2R and TYMP expression, were differentially affected by the venom fractions, further elucidating the specific growth factor and receptor interactions targeted by the venom components [29,30,31]. These findings highlight the distinct molecular mechanisms by which HMM and LMM fractions regulate osteoclastogenesis and immune responses.

Similar modulatory effects of snake venom-derived peptides on osteoclast function have been reported in previous studies [32], supporting the therapeutic potential of these components.

### 3.6. Molecular Pathways and Clusters

STRING analysis revealed distinct molecular clusters involved in OC differentiation, fusion, and resorption, highlighting the differential effects of crude venom fractions. The HMM fraction elicited a broad inflammatory response, prominently activating TNF family signaling and NF-kappa-B pathways upstream of the master transcription factor NFATc1 (Table 2). This likely accelerates OC differentiation and maturation by enhancing classical osteoclastogenic signaling cascades driven by inflammatory cues.

Furthermore, HMM modulated networks associated with CTSK (cathepsin K), a critical protease responsible for bone matrix degradation during resorption, suggesting an increased resorptive capacity of mature OCs under the influence of this venom fraction (Table 2). Collectively, these findings highlight the HMM fraction’s role in amplifying both OC differentiation and functional resorption pathways, consistent with a generalized activation of immune and inflammatory responses.

In contrast, the LMM fraction exerted more targeted effects by modulating immune pathways with a particular emphasis on OC precursor fusion. STRING analysis identified a distinct cluster involving CD44 and CD47, key molecules mediating precursor cell adhesion and fusion (Table 2). LMM treatment fine-tuned these fusion-related interactions, promoting controlled multinucleation while avoiding widespread inflammatory activation. This selective modulation suggests that LMM venom components regulate osteoclastogenesis more subtly, potentially maintaining bone homeostasis while minimizing excessive inflammation.

Together, these results underscore the divergent biological roles of HMM and LMM venom fractions in OC regulation: HMM broadly stimulates inflammatory and differentiation pathways centered on NFATc1 and CTSK, whereas LMM selectively modulates precursor fusion via CD44 and CD47. This differential modulation provides valuable insights into venom bioactivity and informs therapeutic strategies aimed at targeting OC function with minimized inflammatory side effects [27,32]. HMM’s broad inflammatory effects may limit its therapeutic utility, whereas LMM’s selective modulation of OC activity without excessive inflammation highlights its potential as a fine-tuning agent for pathological bone resorption and a more suitable candidate for therapeutic development.

Importantly, our data show that neither the crude venom nor its fractions (HMM and LMM) simply kill osteoclasts or block their differentiation. Instead, they selectively affect different aspects of mature OC function—such as increasing *CTSK* levels, which supports collagen breakdown, while reducing *MMP-9* expression. This means the cells remain alive and active, but their activity is fine-tuned rather than fully shut down. This targeted modulation highlights the potential of venom-derived compounds to carefully regulate OC activity without causing harmful side effects.

This exploratory transcriptomic study provides important molecular insights, but further research is needed to identify the specific bioactive components in the venom fractions, assess their selectivity, and confirm their effects in living organisms. It will also be essential to separate their pro-inflammatory effects from direct actions on OCs to develop safe and effective treatments. Importantly, our results show that neither the crude venom nor its fractions simply block osteoclast formation or kill these cells. Instead, they selectively influence OC differentiation, fusion, and bone-resorbing activity while keeping the cells alive and transcriptionally active. This precise modulation suggests that venom-derived compounds could be valuable tools for controlling excessive bone resorption, offering a promising path toward new therapies with fewer side effects than current anti-resorptive drugs.

Table 2 summarizes the modulation of key OC regulatory nodes—NFATc1, CTSK, and CD44/CD47—by crude *Bothrops moojeni* venom fractions. STRING network analysis revealed that the HMM fraction broadly activates inflammatory and osteoclastogenic pathways, while the LMM fraction selectively modulates precursor fusion without inducing excessive inflammation. These findings highlight the distinct roles of HMM and LMM components in influencing OC differentiation, function, and fusion, with implications for the development of therapeutic strategies targeting osteoclast-mediated bone diseases.

## 4. Conclusions

**Figure 6:**
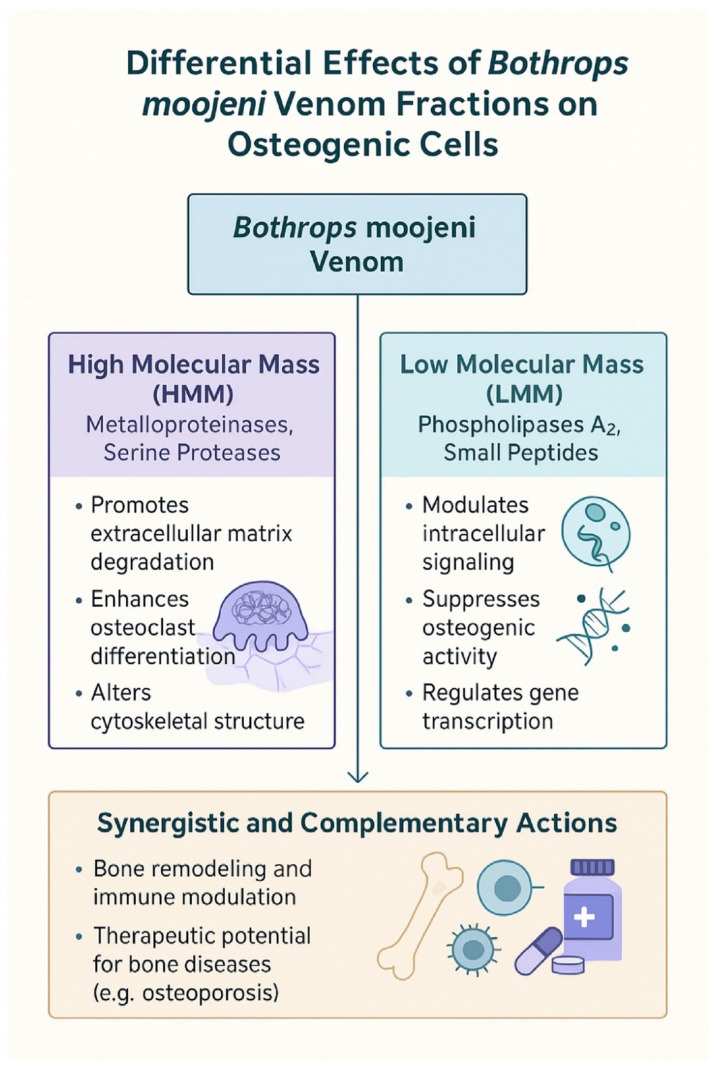
Differential and synergistic effects of *B. moojeni* venom fractions on osteogenic cell function. This diagram summarizes the distinct and complementary actions of HMM and LMM fractions derived from *B.moojeni* venom on osteogenic cells. HMM components, primarily metalloproteinases and serine proteases, promote extracellular matrix degradation, enhance osteoclast differentiation, and alter cytoskeletal structure. In contrast, LMM components, including phospholipases A_2_ and small peptides, modulate intracellular signaling, suppress osteogenic activity, and regulate gene transcription. Together, these venom fractions exhibit synergistic and complementary effects on bone remodeling and immune modulation, highlighting their potential therapeutic applications in bone diseases such as osteoporosis.

These findings underscore the therapeutic potential of *B. moojeni* venom and its fractions in managing bone disorders. By maintaining continuous exposure to crude venom, HMM, and LMM fractions throughout OC differentiation, we were able to capture their cumulative effects on every stage of OC development—from early commitment and fusion to terminal maturation. Crude venom, with its broad-spectrum activity, markedly altered differentiation, fusion, and resorption pathways, indicating promise for conditions driven by excessive bone loss, such as osteoporosis. The HMM fraction—enriched in metalloproteinases and phospholipases—potentiated inflammatory and osteoclastogenic signaling, reflecting its pro-inflammatory influence on bone turnover and suggesting caution in diseases with elevated OC activity. In contrast, the LMM fraction—comprising disintegrins, knottins, and other small peptides—selectively modulated cytokine networks and osteoclastogenesis without triggering widespread inflammation, highlighting its potential as a finely tuned therapeutic agent. Continuous venom pressure throughout differentiation ensured that these effects accumulated and manifested at each developmental stage, lending confidence that the observed gene expression and functional changes are robust and sustained (Figure 6). Future work to isolate and characterize the individual bioactive components responsible will be critical for transforming these fractions into precise, targeted treatments for osteoporosis, rheumatoid arthritis, and other inflammatory bone diseases. Moreover, while the present study provides important functional insights, it lacks direct evidence linking specific enzyme classes (e.g., identified via proteomics) to the observed effects, and detailed proteomic data from these venom fractions is forthcoming.

## 5. Materials and Methods

### 5.1. Isolation of Human Peripheral Blood Mononuclear Cells (PBMCs) and Their Differentiation into OCs

The PBMCs were isolated using the density gradient centrifugation method with Ficoll-Paque (density 1.077 g/mL, Sigma-Aldrich®, St. Louis, MO, USA). Approximately 20 mL of blood was collected from healthy male volunteers, aged 25 to 40 years, via venipuncture in the cubital fossa. The research followed the guidelines established by Plataforma Brasil and received approval from the Butantan Institute’s Ethics Committee (CEP 1,806,596, dated 11 May 2016). All participants signed a consent form authorizing the use of their samples. This authorization was documented in writing through a consent term developed by the laboratory itself for internal purposes, as the ethics committee does not mandate this form. Sample collection occurred between 3 May 2019 and 7 June 2023, with monthly intervals and different donors participating throughout the period. The blood was diluted 1:1 with saline solution (0.9%) and placed in a conical tube containing Ficoll–Paque at a 1:3 ratio. The sample was then centrifuged at 400× *g* for 20 min without acceleration. Following centrifugation, the cells were washed twice with saline solution and resuspended in 1 mL of differentiation medium consisting of α-MEM (Thermo Fisher Scientific, Waltham, MA, USA), supplemented with 10% fetal bovine serum (FBS) ((LGC Biotecnologia, Granja Viana, Cotia, SP, Brazil), 25 ng/mL of human M-CSF (R&D Systems, Minneapolis, MN, USA), 50 ng/mL of human RANKL (R&D Systems, Minneapolis, MN, USA), 5 ng/mL of human TGF-β1 ((R&D Systems, Minneapolis, MN, USA)), and 1 µM dexamethasone (Sigma-Aldrich^®^, St. Louis, MO, USA)). An aliquot of the cell suspension was mixed with an equal volume of Trypan Blue for viability assessment, and the number of viable cells was counted under a light microscope using a Neubauer chamber.

For OC differentiation assays, 6 × 10^5^ PBMCs were plated onto a 1.9 cm^2^ surface area of a 96-well cell culture plate (Corning Incorporated, Costar, Kennebunk, ME, USA) in 200 µL of differentiation medium. The cultures were maintained with media changes twice a week, replacing 50% of the medium volume, over a 15-day period [8]. The crude venom, HMW (high molecular weight), and LMW (low molecular weight) fractions were added at concentrations of 5 µg/mL, 5 µg/mL, and 1 µg/mL, respectively, on days 4, 7, 11, and 14 of PBMC plating. Dose-response experiments (5, 1, and 0.5 µg/mL) for crude venom, HMM, and LMM fractions were performed based on previously published data. Final concentrations were selected to optimize transcriptional modulation while preserving cell viability, considering osteoclast formation, morphology, resorptive activity, and viability [8].

### 5.2. Model Clarification

In this model, PBMCs are used as the source of OC precursors. The culture conditions, supplemented with M-CSF and RANKL, promote OC differentiation while also allowing for the presence of other monocyte-derived cell types, such as macrophages. This mixed cell population mirrors the complexity of the OC niche in vivo, where OCs interact with various cell types, including macrophage-like cells. As a result, RNA-Seq analysis primarily captures the transcriptional signature of OCs, but it also reflects signals from unfused mononuclear cells or macrophage-like populations. This characteristic of the model is beneficial, as it more accurately represents the cellular interactions and environment that OCs experience in their natural niche, providing a more comprehensive view of OC biology.

### 5.3. Determination of Non-Cytotoxic Concentrations of Bothrops moojeni Venom and Its Fractions for Osteoclast Precursor Viability Assays

The non-cytotoxic concentrations of *B. moojeni* venom and its fractions suitable for osteoclast precursor assays were established based on findings from a previous study, which also detailed the venom’s purification and source [8]. Crude venom was obtained from adult *B. moojeni* specimens maintained in the biobank of the Center of Excellence for the Discovery of Molecular Targets (CENTD). Venom was pooled from six adult *Bothrops moojeni* snakes (three males and three females), all raised under identical controlled conditions, to obtain a representative sample of the species. The venom (10.0 mg/mL) was fractionated using a 10 kDa molecular mass cut-off membrane to yield high- (HMM) and low-molecular-mass (LMM) fractions. The efficiency and composition of the separated venom fractions were confirmed by mass spectrometry (Supplementary Material S1).

Cell viability assays confirmed that concentrations of 5 µg/mL for both the crude venom and HMM fraction, and 1 µg/mL for the LMM fraction, did not result in significant cytotoxicity to osteoclast precursors. These concentrations were selected to ensure cell viability while enabling functional analyses, thus allowing for a comprehensive evaluation of the venom’s effects on OC differentiation and activity [8,10].

### 5.4. Total RNA Isolation

For RNA isolation, OCs were harvested using a cell scraper on day 15 of differentiation. The total RNA isolation was performed by first homogenizing the cultured cells using a 100–1000 μL pipette tip with 100 μL of chloroform (Merck, Darmstadt, Germany). The resulting mixture was incubated at room temperature for 10 min to facilitate phase separation. After incubation, the sample was centrifuged at 13,000 RPM for 15 min at 4 °C, resulting in three distinct phases: an upper aqueous phase containing the RNA, an interphase, and a lower organic phase. The upper aqueous phase was carefully aspirated and transferred to a new 1.5 mL polypropylene tube.

Next, 1 μL of glycogen (Thermo Fisher Scientific, Carlsbad, CA, USA) and 250 μL of isopropyl alcohol (Merck, Darmstadt, Germany) were added to the aqueous phase. The sample was gently inverted ten times to mix, followed by incubation at room temperature for 10 min to allow RNA precipitation. After incubation, the sample was centrifuged at 13,000 RPM for 30 min at 4 °C, and the supernatant was discarded.

To wash the RNA pellet, 1 mL of 90% ethanol was added, and the sample was centrifuged again at 13,000 RPM for 20 min at 4 °C. The supernatant was carefully removed by inversion, and this washing step was repeated three times to ensure thorough removal of contaminants. After the final wash, the RNA pellet was air-dried for 5–10 min to avoid over-drying, which can impede resuspension. Finally, the RNA was resuspended in 100 μL of ultrapure, nuclease-free water. This method effectively isolates high-quality RNA suitable for downstream applications, such as RNA sequencing.

The RNA-Seq experiments were performed with three independent biological replicates (n = 3)

### 5.5. Analysis of Isolation RNA Integrity

Assessing the integrity of isolated RNA is a critical step in pre-sequencing quality control, ensuring the reliability of RNA sequencing results [33]. For this purpose, the Agilent 2100 BioAnalyzer system (Agilent, Carlsbad, CA, USA) was employed. This system utilizes microfluidic chips with gel-based size separation channels under voltage induction, coupled with laser-induced fluorescence, to perform high-throughput analysis of up to 20 samples simultaneously [34].

During the procedure, RNA is stained with an intercalating dye and subjected to an electric field. As the RNA migrates through the chip, the fluorescence emitted by the 28S and 18S ribosomal RNA bands is detected by a sensor. The fluorescence intensity of these bands is then used to calculate the areas of the 28S and 18S peaks. The ratio of the 28S to 18S band areas (28S/18S) is determined, and this ratio is analyzed using algorithms based on regression models that have been experimentally validated by the manufacturer. These calculations generate the RNA integrity number (RIN), a value that ranges from 0 (indicating the lowest quality) to 10 (indicating the highest quality).

For RNA-Seq applications, samples with a RIN value greater than 7.0 are considered suitable for sequencing, as they indicate high-quality RNA with minimal degradation [35]. This ensures that the RNA used for sequencing is of sufficient quality to yield reliable and reproducible results.

### 5.6. Library Preparation and RNA Sequencing

The isolated RNA was submitted to CD Genomics (Shirley, New York, NY, USA), a global leader in sequencing services with over a decade of expertise, for RNA-Seq analysis. This analysis focused on the sequencing of messenger RNAs (mRNAs), which encode protein-coding genes. To prepare the cDNA libraries, Illumina sequencing adapters were ligated to both ends of the cDNA fragments: RA5 (5’-GTTCAGAGTTCTACAGTCCGACGATC-3’) to the 5’ end and RA3 (5’-AGATCGGAAGAGCACACGTCT-3’) to the 3’ end. The libraries were then sequenced using the Illumina HiSeq PE150 platform (Illumina, San Diego, CA, USA), which supports paired-end sequencing for both mRNAs and long non-coding RNAs (lncRNAs).

The resulting sequencing data, provided in FASTQ format, were securely transferred via encrypted cloud storage to the Genetics Laboratory at the Butantan Institute. There, the bioinformatics analysis was carried out using a standardized internal pipeline, developed in accordance with best practices for differential gene expression analysis as outlined by [36]). This ensured the generation of high-quality, reproducible transcriptomic data for downstream analysis.

### 5.7. Sequencing Quality Control

The raw sequencing data in FASTQ format were initially subjected to quality control analysis using FastQC software (version 0.11.9), available at https://www.bioinformatics.babraham.ac.uk/projects/fastqc/ (Accessed on 15 January 2019). This tool evaluates several parameters to assess the quality of sequencing data. Among the key metrics reviewed were (i) the per-base sequence quality, which reflects the Phred score for each nucleotide and indicates the likelihood of base calling errors; (ii) the per-sequence quality scores, which summarize the average Phred score across entire reads and provide an overall quality assessment; (iii) the GC content, which was examined for balance in accordance with Chargaff’s rule and the base-pairing principles established by Watson and Crick in 1953, as significant deviations may suggest contamination or amplification bias; and (iv) the sequence length distribution, which helps verify the uniformity of fragment sizes. The Phred quality score (Q) is a logarithmic measure of base call accuracy and is calculated using the formula Q = –10 × log_10_ (E), where E is the probability of an incorrect base call. Accordingly, a Phred score of 20 corresponds to a 1% error rate, indicating 99% confidence in base accuracy. As shown in Table 3, all samples analyzed in this study exhibited Phred scores above 20, confirming the high quality of the sequencing data and their suitability for downstream analysis.

### 5.8. Mapping and Read Counting

Mapping represents a crucial step in the RNA-Seq workflow, as it enables the identification of expressed transcripts and facilitates downstream differential expression analysis. While several alignment tools are available, recent benchmarking studies have demonstrated that the Spliced Transcripts Alignment to a Reference (STAR, version 2.7.8a; available at https://www.biostars.org/p/428681/ (Accessed on 17 January 2019).) consistently outperforms other aligners for RNA-Seq data. STAR is particularly well-suited for this purpose due to its high accuracy in detecting splice variants and its implementation of soft-clipping techniques to remove residual adapter sequences and unaligned bases, thereby enhancing the overall alignment precision [37].

Based on these advantages, the STAR aligner was selected for the present study to map the RNA-Seq reads using the human genome reference GRCh38.p13 and the corresponding GTF annotation file (version 103), both obtained from the ENSEMBL project (http://ftp.ensembl.org/pub/release-103/fasta/homo_sapiens/dna/)(Accessed on 20 January 2019). The Gene Transfer Format (GTF) file facilitated transcript-level annotation, which was used for read quantification.

Following alignment, the resulting BAM (Binary Alignment Map) files underwent quality control using MultiQC (version 1.13; https://multiqc.info/) (Accessed on 25 January 2019), which integrates multiple QC metrics into a single comprehensive report. Each sample achieved a minimum of 10 million reads mapped to the reference genome—a threshold widely regarded as sufficient for robust gene expression analysis [38].

Subsequently, gene-level quantification was performed using the Rsubread Bioconductor package (https://subread.sourceforge.net/ (Accessed on 05 February 2019)), as developed by [39]. Specifically, the featureCounts function was used to assign reads to genomic features based on the aligned BAM files, producing a gene expression matrix that served as the foundation for downstream differential expression analysis.

### 5.9. Normalization and Obtaining the Transcriptome

Following the generation of gene expression matrices, count normalization was performed using the FPM (Fragments Per Million mapped fragments) method, implemented through the DESeq2 package (version 1.36.0; https://bioconductor.org/packages/release/bioc/html/DESeq2.html (Accessed on 20 February 2019), as described by Love et al. This normalization approach adjusts gene counts based on the total number of mapped reads per sample, allowing for accurate comparison of gene expression levels across samples with varying sequencing depths. Differentially expressed genes (DEGs) were identified using DESeq2, with thresholds set at fold change ≥ 2 and false discovery rate (FDR) < 0.05.

After normalization, lowly expressed genes were filtered out to enhance the robustness of downstream analyses. This filtering was conducted using Method 3 of the NOISeq package (version 2.40.0; https://www.bioconductor.org/packages/release/bioc/html/NOISeq.html (Accessed on 20 February 2019)), as recommended by [40]. This method applies a proportion-based test analogous to the Wilcoxon test to identify and exclude genes with minimal expression across the dataset, thereby reducing noise and improving the statistical power of subsequent differential expression analysis.

### 5.10. Functional Enrichment Analysis

Functional enrichment analyses were performed to investigate biological processes and molecular pathways using two complementary approaches: gene set enrichment analysis (GSEA) and over-representation analysis (ORA). GSEA identifies statistically significant differences in gene expression patterns (*p* < 0.05) by considering the entire ranked list of genes, while ORA evaluates the enrichment of specific gene sets based solely on the presence or absence of identified genes, without incorporating expression levels. Both analyses were conducted using well-established genomic databases, including the Kyoto Encyclopedia of Genes and Genomes (KEGG; https://www.genome.jp/kegg/) (Accessed on 15 May 2019). Reactome (https://reactome.org/) (Accessed on 20 May 2019), and WikiPathways (https://www.wikipathways.org/index.php/WikiPathways) (Accessed on 25 May 2019). To enhance the statistical robustness of the results, only pathways with a false discovery rate (FDR) below 0.05 were considered significantly enriched, in accordance with the recommendations by [41].

## Figures and Tables

**Table 1:** Comparison of the Effects of HMM and LMM venom fractions on OC biology and signaling pathways.

Pathway/Cluster	HMM Fraction	LMM Fraction
Differentiation	Modulates classical osteoclastogenesis pathways (e.g., RANKL/RANK) and TNF-related signaling.	Fine-tunes TNF signaling; influences NF-kappa-B pathways to a lesser extent.
Fusion	Induces broad immune responses, influencing precursor fusion.	Enhances CD44 and CD47-mediated fusion, emphasizing immune modulation and bone homeostasis.
Resorption	Influences DC-STAMP activity, contributing to OC fusion.	Modulates IL-6 receptor pathways, affecting bone resorption.
Osteoclastogenesis and Inflammatory Factors	Increases IL-10 signaling, inhibiting osteoclastogenesis.	Stronger modulation of interferon signaling, affecting osteoclastogenesis regulation.
Anti-Osteoclastogenic Inflammatory Factors	Modulates IL-10 signaling and affects immune responses broadly.	Modulates interferon signaling to regulate osteoclastogenesis.
Inhibitory Pathway	Influences negative regulation of B cell differentiation.	Modulates IL-27-mediated signaling, impacting immune responses and OC function.
Non-Canonical Pathway	Affects thioesterase activity and insulin-like growth factor binding.	Modulates insulin-like growth factor mRNA binding and thioesterase activity.
Canonical Pathway	Strong modulation of MAP kinase activity and NF-kappa-B signaling.	Modulates NF-kappa-B and MAP kinase pathways to a lesser degree.
Overall Impact on OC Biology	Induces broader inflammatory responses and OC differentiation, with a focus on classical signaling.	Targets more specific immune and growth factor signaling, with an emphasis on fine-tuning OC function.

**Table 2:** Key osteoclast regulatory nodesm by crude venom fractions: differential effects of HMM and LMM components.

Regulatory Node	Osteoclast Function	Venom Fraction	Effect on STRING Network and Pathways	Functional Implication
NFATc1	Master regulator of OC differentiation and gene expression	HMM	Enhances NF-kappa-B pathway interactions upstream of NFATc1, promoting inflammatory signaling	Increased OC differentiation and maturation driven by inflammation
CTSK	Bone matrix degradation via proteolytic activity	HMM	Upregulates CTSK-associated networks involved in OC activation and resorption	Elevated bone resorption capacity of mature OC
CD44/CD47	Mediate OC precursor fusion and immune modulation	LMM	Selectively modulates CD44/CD47 interaction cluster, affecting cell adhesion and fusion with minimal inflammation	Precise control of OC precursor fusion and multinucleation without broad immune activation

**Table 3:** Summary of Phred quality scores for RNA-Seq samples demonstrating high sequencing accuracy.

Phred Score (Q)	Error (E)	Accuracy (1 − Error)
10	1/10 = 10%	90%
20	1/100 = 1%	99%
30	1/1000 = 0.1%	99.9%
40	1/10,000 = 0.01%	99.99%
50	1/1,000,000 = 0.001%	99.999%
60	1/10,000,000 = 0.0001%	99.9999%

Source: https://gatk.broadinstitute.org/hc/en-us/articles/360035531872-Phred-scaled-quality-scores (Accessed on 15 January 2019).

## Data Availability

The original contributions presented in this study are included in this article and the Supplementary Materials. Further inquiries can be directed to the corresponding authors.

## Supplementary Materials

The following supporting information can be downloaded at: https://www.mdpi.com/article/10.3390/toxins17070358/s1, Supplementary Material S1: Analisys of proteome of High and low molecular mass of *Bothrops moojeni* venom.
